# Jottings

**Published:** 1909-11-13

**Authors:** 


### JOTTINGS.

Christchurch is to have a new infirmary, which will cost £12,000.

The General Hospital, Nottingham, celebrated its 127th anniversary last week.

The annual sale of work in aid of the funds of Newry General Hospital was held last week.

It is proposed to start an open-air school for tuberculous children at Blencathra, near Carlisle.

The annual street collection in aid of the Hospital Saturday Fund in Belfast realised nearly £1,000.

Runcorn Cottage Hospital has added a new wing, costing over £1,000, which was opened on October 16 by Mr. A. T. Smith.

The new children's ward at the Royal City of Dublin Hospital, Baggot Street was opened last week by the Countess of Pembroke.

Warwick Joint Hospital will have a new convalescent ward and the Board has appointed a sub-committee to discuss ways and means.

The third Pound Day of the Royal Victoria Hospital, Bournemouth, realised £40 odd. Some 4,000 lbs. of groceries were contributed.

The amount raised in the West Bromwich district this year by Hospital Saturday collections was £2,095, a decrease of £111 compared with last year's collection.

The King has sent to the Rev. J. Vincer Minter a donation of 25 guineas towards the expenses of the choir excursion of St. Etheldreda, the Newmarket Union Workhouse Church. The Rev. Vincent Minter is the only workhouse master in England who is also a clerk in holy orders.

The Greenock Eye Infirmary has lost a true and valued old friend through the death of Mr. Anderson Rodger, a wealthy shipbuilder, who 15 years ago founded the institution.

At the West of England Eye Infirmary there has been a very large increase in the number of school children patients treated at the institution, in consequence of the medical inpection of schools.

The Hospitals Committee of the Metropolitan Asylums Board have decided to continue for a further twelve months the experiment of holding morning classes for medical instruction at certain of the hospitals.

The Glasgow Town Council has decided to erect an observation ward at Ruchill Hospital, at an estimated cost of £7,500. The scheme provides a block of ten separate wards accommodating forty-eight patients.

At the annual meeting of the Baschurch Women and Children's Convalescent Home it was reported that the average stay of a patient in the home was 11 weeks 3 days, and the average daily cost per patient 1s. 10d.

The Orsett Guardians have passed a resolution that owing to the increase in the work in the Infirmary no serious operations be performed in the Infirmary unless they are absolutely imperative and the cases irremovable.

It is extremely doubtful if the proposed scheme for the establishment of a rate-supported phthisis institution for the county of Surrey, for the benefit of patients in the Poor Law Unions of the county will come to anything. Seven Boards of Guardians have already declined to take further action.

